# Protocol for a multicenter, randomised controlled trial of surgeon-directed home therapy vs. outpatient rehabilitation by physical therapists for reverse total shoulder arthroplasty: the SHORT trial

**DOI:** 10.1186/s40945-021-00121-2

**Published:** 2021-12-10

**Authors:** June S. Kennedy, Emily K. Reinke, Lisa G. M. Friedman, Chad Cook, Brian Forsythe, Robert Gillespie, Armodios Hatzidakis, Andrew Jawa, Peter Johnston, Sameer Nagda, Gregory Nicholson, Benjamin Sears, Brent Wiesel, Grant E. Garrigues, the SHORT Trial Investigators, Christopher Hagen, Insup Hong, Marcella Roach, Natasha Jones, Kuhan Mahendraraj, Evan Michaelson, Jackie Bader, Libby Mauter, Sunita Mengers, Nellie Renko, John Strony, Paul Hart, Elle Steele, Amanda Naylor, Jaina Gaudette, Katherine Sprengel

**Affiliations:** 1 Department of Physical and Occupational Therapy, Duke University Health System, 3475 Erwin Rd, Durham, NC 27705 USA; 2 Department of Orthopaedic Surgery, Duke University Medical Center, 3475 Erwin Rd, Durham, NC 27705 USA; 3 Department of Orthopedics, Geisinger Medical Center, 100 N Academy Ave, Danville, PA 17822 USA; 4 Duke Clinical Research Institute, Duke University, 200 Morris Street, Durham, NC 27701 USA; 5 Duke Department of Population Health Sciences, Duke University, 215 Morris St, Durham, NC 27701 USA; 6 Department of Orthopaedic Surgery, Rush University Medical Center, 1611 W. Harrison St., Suite 400, Chicago, IL 60612 USA; 7 Department of Orthopaedic Surgery, University Hospitals Cleveland, 11000 Euclid Ave, Cleveland, OH 44106 United States; 8 Western Orthopaedics, 1830 Franklin St, 450, Denver, CO 80218 USA; 9 Boston Sports and Shoulder Center, 40 Allied Dr., Suite 102, Dedham, MA 02026 USA; 10 Centers for Advanced Orthopaedics, 25500 Point Lookout Road, Leonardtown, MD 20650 USA; 11 Anderson Orthopaedic Clinic, 2445 Army Navy Drive, Arlington, VA 22206 USA; 12 MedStar Orthopaedic Institute, 3800 Reservoir Road Northwest, Washington, DC 20007 USA

**Keywords:** Reverse total shoulder arthroplasty, Rehabilitation, Home therapy, Physical therapy, Shoulder arthritis, Shoulder, Clinical outcomes, Patient reported outcomes, Complications, Costs

## Abstract

**Background:**

Reverse total shoulder arthroplasty (RTSA) has emerged as a successful surgery with expanding indications. Outcomes may be influenced by post-operative rehabilitation; however, there is a dearth of research regarding optimal rehabilitation strategy following RTSA. The primary purpose of this study is to compare patient reported and clinical outcomes after RTSA in two groups: in one group rehabilitation is directed by formal, outpatient clinic-based physical therapists (PT group) as compared to a home therapy group, in which patients are instructed in their rehabilitative exercises by surgeons at post-operative appointments (HT group). Secondary aims include comparisons of complications, cost of care and quality of life between the two groups.

**Methods:**

This randomised controlled trial has commenced at seven sites across the United States. Data is being collected on 200 subjects by clinical research assistants pre-operatively and post-operatively at 2, 6, and 12 weeks, 6 months, 1 and 2 year visits. The following variables are being assessed: American Shoulder and Elbow Surgeons (ASES), pain level using the numeric pain scale, the Single Assessment Numeric Evaluation (SANE) score, and shoulder active and passive range of motion for analysis of the primary aim. Chi square and t-tests will be used to measure differences in baseline characteristics of both groups. Repeated measures linear mixed effects modeling for measurement of differences will be used for outcomes associated with ASES and SANE and scores, and range of motion measures. Secondary aims will be analyzed for comparison of complications, cost, and quality of life assessment scores using data obtained from the PROMIS 29 v. 2, questionnaires administered at standard of care post-operative visits, and the electronic health record. Subjects will be allowed to crossover between the PT and HT groups, and analysis will include both intention-to-treat including patients who crossed over, and a second with cross-over patients removed, truncated to the time they crossed over.

**Discussion:**

RTSA is being performed with increasing frequency, and the optimal rehabilitation strategy is unclear. This study will help clarify the role of formal physical therapy with particular consideration to outcomes, cost, and complications. In addition, this study will evaluate a proposed rehabilitation strategy.

**Trial registration:**

This study is registered as NCT03719859 at ClincialTrials.gov.

**Supplementary Information:**

The online version contains supplementary material available at 10.1186/s40945-021-00121-2.

## Background

Growing acceptance of the expanding indications and the success of reverse total shoulder arthroplasty (RTSA) has led to a dramatic increase in shoulder arthroplasty in the United States [1]. Between 2011 and 2017 the number of RTSA surgeries in the United States increased 191.3% with 63,845 procedures performed in 2017 [2]. Initially used only for cuff tear arthropathy, [3, 4] RTSA is now frequently employed for a wider variety of shoulder pathologies including proximal humerus fractures, rheumatoid arthritis, osteoarthritis (OA), tumors, avascular necrosis, and massive rotator cuff tears without arthritis [3, 5–8]. The RTSA prosthesis is effective at providing improved active motion and function due to the semi-constrained design. This design substitutes for the centering effect of the deficient rotator cuff and allows the deltoid to elevate or abduct the arm with fixed-fulcrum kinematics [9, 10]. Many factors are hypothesized to influence the potential for a successful outcome following RTSA: proper patient selection, surgeon expertise, prosthetic design, surgical technique and approach, and post-operative rehabilitation [11–13]. Whilst prior researchers have explored the effect of surgical technique, type of prosthesis and surgical indications on outcome following RTSA, [14] there is limited literature on the influence of post-operative rehabilitation on outcomes. The surgical procedure is often reserved for an elderly population who are at least 65 years of age, [15] and a younger age is a risk factor for post-operative complications [16].

Dating back to the work of Hughes and Neer, [17] a proper rehabilitation strategy has been felt to be imperative following shoulder arthroplasty. Early range of motion in a protected and graduated way has been proposed to avoid stiffness and muscle atrophy whilst also protecting healing tissues, and avoiding complications such as instability and stress fractures [17–26]. There are multiple published rehabilitation protocols for both anatomic total shoulder arthroplasty (ATSA) and RTSA, which include thorough biomechanical rationales [17–26]. However, as Bullock et al. reported in a recent systematic review of proposed rehabilitation guidelines, there is nominal consensus regarding rehabilitation strategies, and there is a need for high-quality prospective research [27]. Currently, there are only four published studies on therapy after ATSA [24, 28–30], and two published studies evaluating the rehabilitative strategy for RTSA [31, 32]. One prospective ATSA [28] and two prospective RTSA [31, 32] studies report on the impact of immediate versus delayed therapy, and all conclude that there is overall no significant difference in clinical and patient reported outcomes. Three retrospective studies [24, 29, 30] report on outcomes for therapy after ATSA, with one study concluding that home therapy directed by the surgeon had favorable outcomes compared to formal physical therapy [29]. A retrospective case series demonstrated successful range of motion and patient reported outcomes following ATSA when rehabilitation was conducted at home using web-based exercises generated and provided by the surgeon [30]. There are no published randomised controlled trials comparing home therapy to formal physical therapy following RTSA.

There is nominal consensus on the optimal therapy protocol following RTSA, or on who should instruct and supervise rehabilitation [33]. The only consensus is that therapy is felt to play an important part in RTSA outcomes and that there is a need for research to guide this clinical question [27, 33]. Authors universally note the unsettling discrepancy between “doing nothing” [18] and a formal protocol involving multiple outpatient or even inpatient visits. This discrepancy in service provision suggests a need for a well-designed randomised trial [27, 33]--especially given the cost associated with the latter strategy [18, 29].

We aim to compare outcomes for formal, clinic-based physical therapy (PT group) to home therapy, in which patients are provided with instructions from surgeons at post-operative appointments (HT group) by using a superiority, randomised design [34]. Findings will aid in determining the potential role of formal PT in the RTSA recovery process, and secondarily, will evaluate a formal RTSA rehabilitation protocol, which is standardized for the trial [20].. Findings from the study may assist in evaluating the financial benefit or burden of formal PT in the RTSA recovery process and determine the resource necessity of PTs in a traditional clinical environment. We hypothesize that the PT group will demonstrate superior clinical and patient reported outcomes, significantly quicker levels of recovery, and significantly higher levels of overall recovery at our long-term outcomes capture when compared to the HT group. Secondary aims of this project include analysis of the incidence of complications between the two groups, specifically acromial stress fractures and prosthetic instability events, which we hypothesize will be higher in the PT group; and cost of care which we hypothesize will be higher in the PT group.

## Methods

### Study design

This is a multicenter, prospective, randomised controlled trial with parallel groups evaluating patients who have RTSA at one of the participating sites by a shoulder fellowship-trained American Shoulder and Elbow Surgeons member. These surgeons have specialized training in shoulder surgery including the RTSA procedure. This protocol was created using the Standard Protocol Items, Recommendations for Interventional Trials (SPIRIT) reporting parameters [35]. A checklist demonstrating compliance with the SPIRIT parameters is included as supplemental material. This study was prospectively registered as NCT03719859 at ClincialTrials.gov. The study is externally funded by the Orthopedic Research and Education/The Aircast Foundation grant number 18–058. The role of this funding source is solely financial and not influential or contributory to the design of the protocol or interpretation of results.

### Study setting

The lead institution is Rush University Medical Center; and 6 additional participating sites include: University Hospitals Cleveland Medical Center; Western Orthopaedics; Boston Sports and Shoulder Center; Centers for Advanced Orthopedics; Anderson Orthopaedic Clinic; and MedStar Orthopaedic Institute.

### Research ethics approval

A full non-expedited Institutional Review Board approval of this study was provided on 8.17.20 (ORA: 18082102-IRB01) at the lead institution (Rush University Medical Center) including a patient informed consent form. A full Institutional Review Board approval also is required for each participating site. The approved informed consent form which is being used at the lead research site (Rush University Medical Center) is included in the Supplemental materials. Any changes to the research protocol will need approval by the IRB for all sites.

### Eligibility criteria/subjects

Participants are identified as potential subjects for this study if they are over 50 years of age, under the care of one of the participating surgeons, and elect to have RTSA for rotator cuff tear arthropathy, massive irreparable rotator cuff tear, or primary osteoarthritis. Subjects are excluded from study participation for the following reasons: history of prior ipsilateral open shoulder surgery, non-reverse total shoulder arthroplasty, RTSA for fracture or revision arthroplasty, RTSA with a tendon transfer, discharge to a skilled nursing facility, in-patient rehabilitation placement, or use of home health therapy prior to progressing in recovery, unwilling to make a good faith effort to adhere to their randomly prescribed rehabilitation scheme, unable to speak, read, or write the English language, have cognitive deficits limiting ability to follow direction, unable or unwilling to be randomised due to financial or personal constraints, or have inability to attend physical therapy (i.e. transportation or financial limitations). Subjects who meet the inclusion criteria are identified at clinical appointments with participating surgeons, and clinical research assistants discuss the study process and review the voluntary informed consent process. At this appointment the following variables are obtained as pre-operative baseline values: American Shoulder and Elbow Surgery (ASES) score, Numeric Pain Rating Scale (NPRS) level, Single Assessment Numeric Evaluation (SANE) score, active range of motion (AROM), passive range of motion (PROM), and Patient-Reported Outcomes Measurement Information System (PROMIS) score. Upon voluntary consent to participate, clinical research assistants employ computer based Research Electronic Database Capture (REDCap) (Nashville, TN) [36] to perform the allocation sequencing of subjects to either the PT or HT group. The randomization schedule is a randomized block design stratified by site using a 1:1 treatment allocation in blocks of size 8.. Patients are encouraged to remain within the group to which they are randomly assigned for the duration of the study, however, if the patient, surgeon, or therapist has concern regarding patient safety, compliance or outcome due to the group assignment, cross-over to the other group will be allowed. The research plan is for approximately a 15% crossover rate based on prior research which planned for 10%, but actually ended up with 30% crossover [37]. The reason for cross-over will be recorded in the electronic health record and reported in the study result.

Patients who participate in this project are offered $25 gift cards at the 6 month office visit to thank them for their participation and encourage study retention.

All study patients are treated with an RTSA using a deltopectoral approach. Different implant systems are being utilized at the discretion of the treating surgeon based on their preferred implant. To assess for potential confounding variables, implant characteristics which might affect range of motion, stability, and potential for acromial stress fractures are captured including: implant manufacturer, implant model, glenosphere center of rotation from the glenoid face (recorded in millimeters), glenosphere diameter, and polyethelene liner thickness.

All patients in the study attend post-operative appointments with their surgeon at 2 and 6 weeks, 3 months, 6 months, 1 and 2 years according to standard of care. These are the same intervals at which data is collected by clinical research assistants.

### Interventions

#### Rehabilitation management

##### Physical therapy and home therapy groups

Prior to discharge from the hospital or surgical center, all subjects in both study groups (PT or HT) receive the same phase 1 exercise instruction by occupational or physical therapists. Patients in the PT and HT groups follow the rehabilitation guidelines proposed by Boudreau et al. [20] which are detailed in Table 1.

**Table 1:** Rehabilitation guidelines for reverse total shoulder arthroplasty

PHASE	PRECAUTIONS AND GUIDELINES	GOALS	EXERCISES	CRITERIA TO ADVANCE TO NEXT PHASE
1 (post-op day 1-2 week)	Sling 24/7 (remove for grooming and HEP-3 5x/day) Avoid hand behind back, and reaching cross body Keep arm anterior frontal plane “always see elbow” No shoulder AROM No submersion in water No weight bearing on shoulder	Protect prosthesis from dislocation Prevent infection Promote distal circulation Proper sling fit PROM: 120 elevation and 30 ER	Pendulum Active elbow, wrist and hand, scapular retraction Passive elevation to 90-120 deg in scapular plane Passive ER to 30 deg inscapular plane	Pain less than 3/10 with PROM Healing incision without signs of infection Clearance by MD after radiograph assessment at 2 week check up
2 (3-6 wks)	Sling only in community Use of operative arm allowed for basic ADLs with elbow beside waist – nothing heavier than a coffee cup. No active reaching from shoulder May submerge in water (eg pool or hot tub) after 4 weeks Continue no shoulder extension, hand behind back, cross body or weight bearing	Passive elevation to 120; ER to 30 Able to fire all heads of deltoid Pain < 3/10	Discontinue elbow, wrist, and hand exs since using arm of ADLs Continue pendulum, scapular retraction, PROM for elevation and ER 120/30 in scapular plane ADD: deltoid isometrics for all heads (avoid extension beyond frontal plane) Reverse pendulum at 90 deg elevation in supine	Passive elevation to 120 and ER to 30 degrees Able to fire all heads of deltoid without pain Able to place and hold arm at 90 deg in supine (balanced position)
3 (6-12 wks)	Discontinue sling Motion recovery without excessive force Advance arm use in ADLs gradually May begin hand behind back gently NO Upper Body Ergometer due to repetitive loading of deltoid on acromion	Optimize PROM Develop AROM to match available PROM Establish dynamic stability of shoulder with deltoid and parascapular strengthening, as well as any rotator cuff remaning	Active forward elevation progression: supine to inclined to vertical, short to long lever arm (bent to straight elbow) Active ER/IR with arm at sideTheraband scapular retraction IR behind back gently	AROM when upright equals PROM in supine No pain Need higher level demand than ADL functions (eg sport or work)
4 (12+ wks)	Avoid heavy lifting and overhead sport Avoid heavy pushing May lift light weights for deltoid but not to exceed 3 lbs NO Upper Body Ergometer	Functional demands for work and/or sport achieved Gradual increase in deltoid and parascapular muscle strength Painfree	Weights for deltoid up to 3 lbs max, using short lever arm (bent elbow) for middle deltoid raise Theraband progression for scapular muscles, including serratus anterior punches Gentle end range stretching in all planes as part of a daily lifelong routine	NA

##### Physical therapy intervention group

Subjects in the PT group will begin out-patient clinic-based rehabilitation 4–5 days after discharge from the hospital and continue in therapy approximately once a week for three months. Physical therapists will employ a pragmatic approach within the rehabilitation guidelines summarized in Table 1. Physical therapists will determine the optimal exercises for each patient within the specified guidelines for each phase of recovery. The exercises may vary amongst subjects considering patient specific variables such as exercise tolerance and pain response, ease of progressing to next level of challenge, ability to follow directions and perform exercises correctly, and individual goals for rehabilitation. The number of physical therapy visits may vary amongst patients in this group depending on response to therapy. For example, patients with low demand goals for rehabilitation and/or those who tolerate therapy well and advance easily may need less PT visits than patients with high demand goals and/or those who don’t tolerate therapy well and progress more slowly.

##### Home therapy group

Patients continue to perform the exercises they learned in the hospital prior to discharge until they follow-up with the surgeon per the standard of care approximately 2 weeks after surgery. Rehabilitation exercises and activity guidelines will be advanced for the HT group by the treating surgeon using pre-printed exercise handouts which follow the rehabilitation guidelines in Table 1. The handouts will not vary amongst subjects for consideration of patient specific variables. All subjects will receive the same exercise progression at the 2 week, 6 week, and 3 month appointment.

Additional physical therapy following the RTSA procedure (not in the PT or HT group) will not be allowed. Other physical care such as chiropractic or massage intervention for conditions not related to the RTSA procedure will be allowed upon approval by the treating surgeon.

### Data collection

#### Descriptive information

Patient age at time of surgery, sex, height, weight, hand dominance, laterality of surgery, reason for RTSA (cuff tear arthropathy, primary osteoarthritis, or massive rotator cuff tear with pseudoparalysis), history of prior ipsilateral shoulder surgery (e.g. rotator cuff repair), history of prior ipsilateral shoulder fracture, and history of prior ipsilateral shoulder dislocation, and the type of RTSA prosthesis employed in procedure will be obtained from the medical record. Final data will be kept confidentially at the lead institution.

#### Primary outcomes

The American Shoulder and Elbow Surgeons (ASES) questionnaire is a standardized method of evaluating shoulder pain and function. This instrument is a 100-point scale which assesses pain (50 points) and activities of daily living (50 points), and has demonstrated good reliability/validity following shoulder arthroplasty [38]. The ASES score clinically important change following shoulder arthroplasty is 9 to 10 points [11, 39].

Pain level is monitored using the 0–11 numeric pain rating scale (0 = no pain; 10 = worst pain imaginable) at present, as well as the best and worst rating over the 2 week interval preceding assessment.

The Single Assessment Numeric Evaluation (SANE) score asks a patient the question: “How would you rate your affected joint/region of interest today as a percentage of normal (0% to 100% scale) with 100% being normal?” and has been validated for patients having shoulder arthroplasty [40].

The Patient-Reported Outcomes Measurement Information System (PROMIS-29) version 2 .0 profile assesses pain intensity (0 to 10) as well as seven health domains including physical function, fatigue, pain interference, depressive symptoms, anxiety, social interactions, and sleep disturbance with 4 items per domain. The physical health factor (physical function, pain intensity and interference, and social interactions) and mental health factor (anxiety and depressive symptoms) have been established as valid and reliable [41]. The PROMIS has been determined to be a responsive instrument following RTSA [42].

##### Range of motion

Passive and active scaption, external rotation with the arm at the side (ER0), external rotation at 90 degrees of abduction in the scapular plane (ER90), and active internal rotation (IR) range of motion are measured pre-operatively and at 6 weeks, 3 months, 6 months, 1 and 2 years post-operatively. Active range of motion is measured at the end of the range of motion self-selected by the subject with the instruction to move as far as possible without pain. Passive range of motion is provided by the measurer with the instruction “Please allow me to move your arm as far as possible without increasing pain.”

**Fig. 1:**
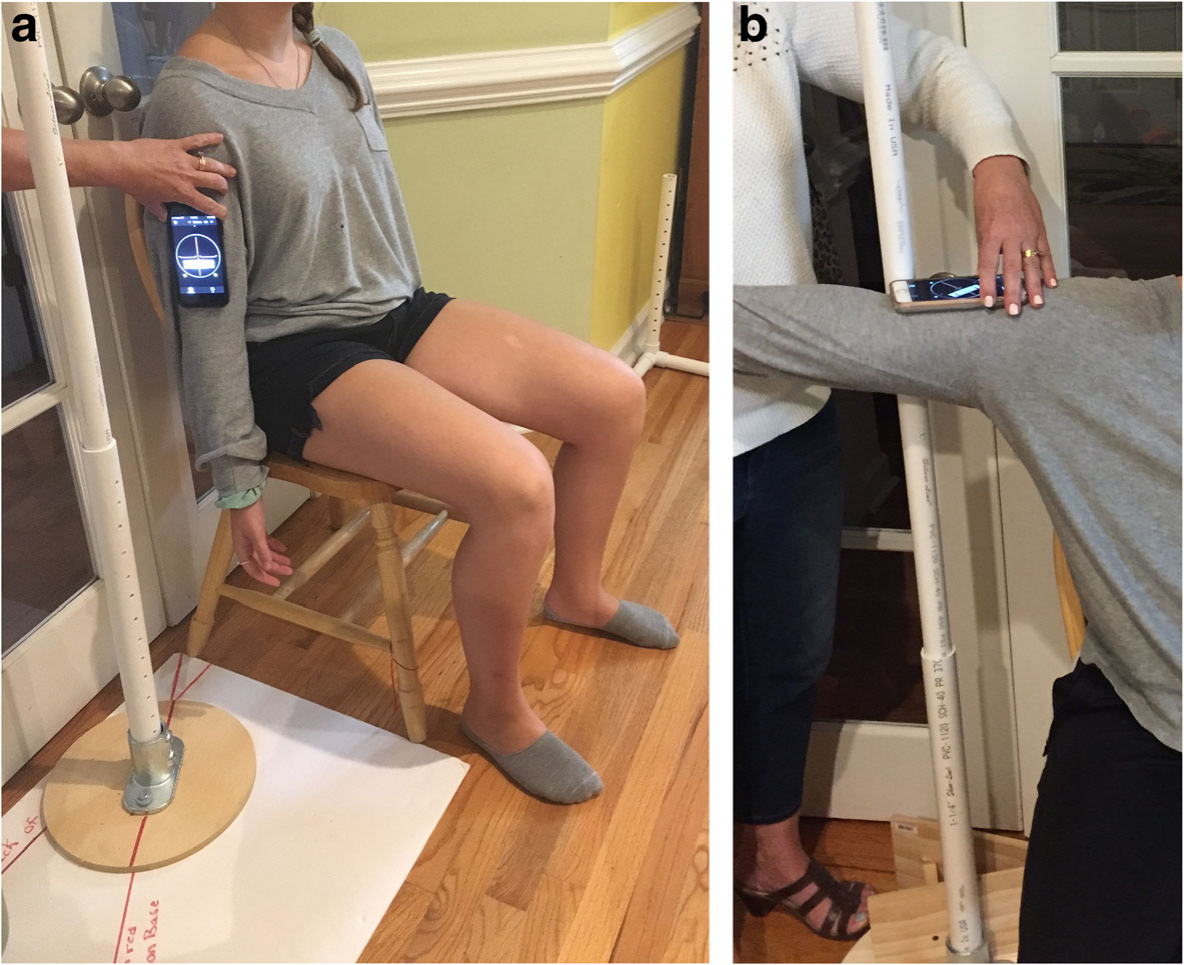
**A**. Start position for measuring scaption. **B**. End position for measuring scaption

**Fig. 2:**
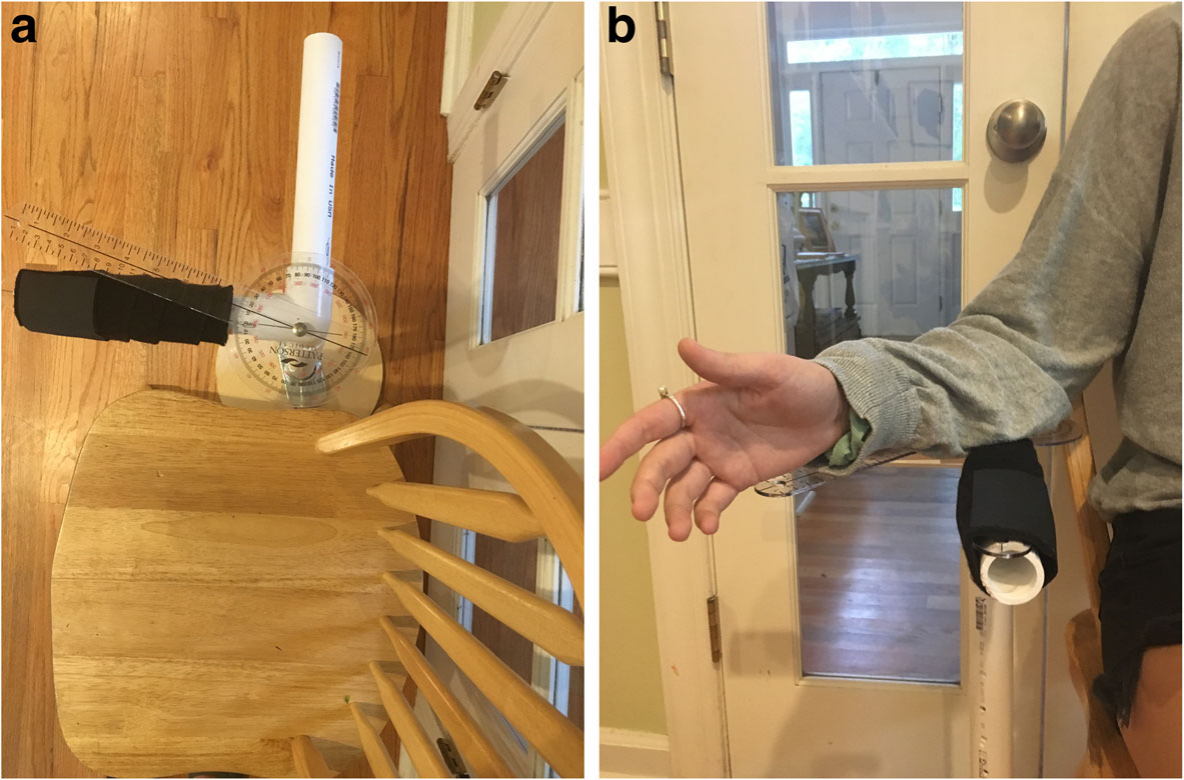
**A**. Set up for measuring external rotation in neutral position with the arm at the side. **B**. Measuring scaption with subject’s arm placed on top of the goniometer

**Fig. 3:**
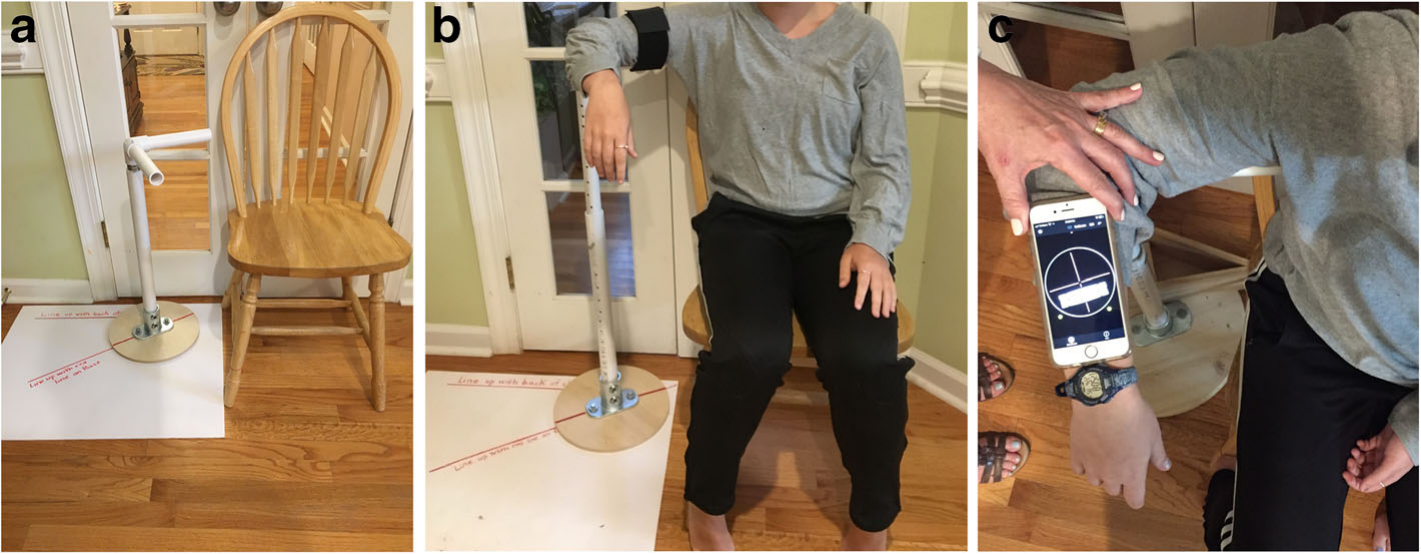
**A**. Set up for measuring external rotation at 90 degrees of abduction. **B**. Patient, and **C** Clinometer positioning for measuring active and passive external rotation at 90 degrees of abduction

Standardization of the method of measuring ROM was deemed necessary due to the multi-center nature of this trial employing several research assistants to obtain measures. A custom device for guiding scaption (elevation 30 degrees anterior the frontal plane), and a custom device for stabilizing the arm for ER measures were constructed (Figs. 1, 2 and 3). A validated smart-phone inclinometer application (Clinometer, plaincode, www.plaincode.com) is used to measure scaption and ER(90) [43]. A goniometer is used to measure ER(0), and the Constant-Murley method of scoring functional IR is utilized [44].

Scaption is measured by having the subject elevate the arm along a plastic pipe anchored to a base placed 30 degrees anterior the frontal plane (Fig. 1a). The clinometer application is zeroed with the arm at the side prior to scaption and read at the end of the active or passive motion (Fig. 1b). ER (0) is measured by stabilizing one arm of a plastic goniometer to a forward facing plastic pipe placed beside the subject, with the axis of the goniometer under the subjects’ elbow (Fig. 2a). The mobile arm of the goniometer is aligned under the ulnar side of the forearm at the end of the ER (0) motion (Fig. 2b). ER (90) is measured with the upper arm stabilized to a plastic pipe at shoulder height oriented in the scapular plane (assumed at 30 degrees anterior the frontal plane), and the participant rotates the forearm up from a second plastic pipe which is forward facing (Fig. 3a). The Clinometer is zeroed at the horizontal, and read at end range of ER (90) (Fig. 3b and c). Internal rotation is only measured actively, not passively, by observing the highest anatomic landmark achieved using the Constant-Murley scoring method [44].

#### Range of motion reliability study

Due to the novel range of motion testing device employed by researchers at seven test sites in the SHORT Trial, a feasibility and reliability study to determine the reproducibility of measures was deemed necessary. Inter-tester reliability for the standardized methods of obtaining range of motion measures was established by having 10 different measurers of varied experience (medical and non-medical backgrounds) assess each of the motions (scaption, ER (0), ER (90), and IR) on three different subjects. Active and passive range of motion measurements were obtained three times on each subject, and the means of the three active and three passive trials were used to develop a Pearson inter-tester correlation coefficient (ICC) for each motion. For each of the three trials, subjects moved to a pre-determined range of motion, which was either at the beginning of the range, mid-range, or end range in order to ensure reliability throughout range of motion. Variability due to subjects moving to different points in the range of motion was minimized by having this pre-determined range of motion; measurers were blinded to the pre-set subject range of motion value. The 10 measurers were provided with a training video explaining how to obtain the measures, a detailed training manual, and clinic cards with measurement method summaries and pictures for use as a reference. These materials are the same used to train all clinical research assistants at each participating center in the study. Examination of the data following the first reliability study revealed that the numbers obtained for ER (90) were not reflections of the true range of motion measured, and there was more variability than desired for ER (0). Measures of ER (90) were not accurate because the Clinometer was not being zeroed at the true horizontal, therefore this step was added to the measurement process. The ER (0) variability was attributed to the goniometer being placed on the top of the subjects’ forearm which created difficulty for accurate alignment of the axis of rotation at the elbow. A change in the method was devised to have subjects rest the arm on top of the goniometer stable arm, which was secured to the custom device, and have the subject place the elbow on the goniometer axis of rotation. Following these changes in ROM methods upon completion of the pilot reliability study, a second reliability trial was conducted in the same manner which revealed that the ICCs were considered to be high, [45] reflecting that these methods can be repeated with reliability across participating study sites: scaption 0.98, ER (0) 0.89, ER (90) 0.69, and the active IR was repeated within 2 measurement levels across all subjects.

#### Secondary outcomes

##### Costs and complications data

Total number of visits to the physical therapist for patients in the PT group, compliance with the therapy plan, and complications are obtained from the medical record and the patient questionnaires (Appendix A). Medicare and private payer physical therapy reimbursement data are obtained through the PearlDiver database. Whilst we recognize that “true cost” is a fluid concept in health care, this information, along with calculation of quality adjusted life years from PROMIS-29 v. 2.0 allows us to perform a cost-effectiveness analysis.

Table 2 lists and defines complications tracked as part of the study. We specifically are assessing for the incidence of acromial or scapular spine stress fractures and shoulder dislocation which may be impacted by the rehabilitation strategy, and therefore will be compared between groups. Additional complications including infection, nerve palsy, or other post-operative events related to surgery (eg. periprosthetic fracture) will not be compared between groups. In addition, any unanticipated presentation to a hospital, urgent care, or physician’s office for any reason is also recorded as an adverse event. All complications and adverse events are reviewed by an independent safety monitor.

**Table 2:** Complications tracking chart

Complication	Definition
Acromial or scapular spine stress fracture	Diagnosed clinically with following findings: 1. Sharp pain referred to the acromion/scapular spine worse with deltoid activation; 2. Tenderness with palpation of the acromion/scapular spine.
Dislocation	Radiographically confirmed dislocation of the articulating surfaces.
Infection	“Definite” or “Probable” periprosthetic infection as diagnosed by the ICM criteria [46].
Nerve palsy	Impairment of an ipsilateral upper extremity nerve as detected by loss of sensation or a reduction in motor strength in the distribution of a particular peripheral nerve
Other (related to surgery) Explain	Any other complication related to the study shoulder (eg. prosthetic loosening, mechanical dissociation, periprosthetic fracture)
Other (adverse event, unrelated to surgery) Explain	Unanticipated presentation to a hospital, urgent care, or physician’s office for any reason not categorized above within 90 days post surgery.

Clinical research assistants are not blinded due to the need to facilitate appropriate group assignments therefore they are aware of patient group assignment (PT vs HT). Patients are not blinded to group assignment due to their awareness of the rehabilitation occurring at either a physical therapy clinic in weekly appointments or at routine post-operative appointments with the surgeon. For subjects in the PT group, clinical research assistants arrange physical therapy appointments at the appropriate follow-up and ensures the physical therapist is trained with the protocol. For subjects in the HT group, clinical research assistants facilitate the progression of exercises that are taught by the surgeons at the post-operative standard of care appointments during the first 3 months of follow-up. Surgeons and clinical research assistants are trained in the HT exercise progression provided in the pre-printed exercise sheets. For both groups, clinical research assistants obtain the patient reported outcome measures, range of motion measures, and collect patient data regarding compliance, cost, and complications.

### Treatment fidelity assurance

Fidelity to the therapy program is deemed important as therapists have been shown to demonstrate drift to their own preferred practice of rehabilitation which can introduce variability in outcomes [47]. To help reduce variability attributable to therapists not following the provided rehabilitation protocol, training was provided to a lead therapist at each participating site with a one hour webinar describing the rehabilitation program. These lead therapists serve as a resource for each participating site if questions arise regarding the rehabilitation strategy. Patients who participate in the study may travel from remote locations to the participating surgical sites to receive RTSA. Upon returning to their hometown after surgery, these patients may see therapists other than those at participating sites. In an effort to standardize and optimize fidelity to the formal PT intervention, therapist instruction booklets detailing the rationale of the rehabilitation plan as well as the rehabilitation guidelines are provided to each therapist treating patients in the PT group. The patients in the HT group all receive the same exercise handouts to provide consistency amongst these subjects.

### Participant timeline

**Fig. 4:**
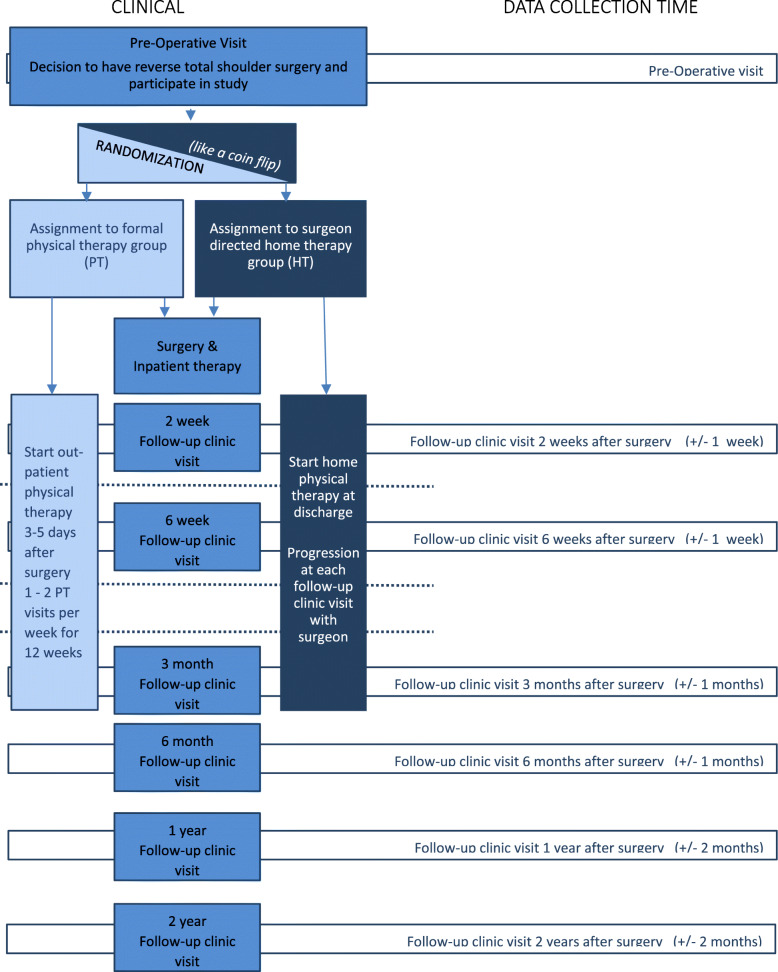
Study Flow Diagram

Subjects are expected to participate in the trial for a total of two years following the date of surgery. ASES score, pain level, SANE score, and PROMIS-29 v. 2.0 scores are collected preoperatively and at standard of care post-operative appointments at 2 week 6 weeks, 3 months, 6 months, 1, and 2 years. Passive and active range of motion is also collected pre-operatively and at all the post-operative appointments with the exception of the 2 week post-operative appointment. A custom patient questionnaire is administered at every standard of care post-operative appointment. The timeline for variable data collection points is summarized in Table 3. Figure 4 illustrates the study flow including enrollment, interventions, assessment and visits. The decision to terminate the trial rests with the principal investigator.

**Table 3:** Outcome measures collected at each timepoint of study

	Pre-op	2 weeks	6 weeks	3 months	6 months	12 months	24 months
**ASES**	Yes	Yes	Yes	Yes	Yes	Yes	yes
**Pain**	Yes	Yes	Yes	Yes	Yes	Yes	yes
**SANE**	Yes	Yes	Yes	Yes	Yes	Yes	yes
**Active ROM**	Yes		Yes	Yes	Yes	Yes	Yes
**Passive ROM**	Yes		Yes	Yes	Yes	Yes	yes
**Study questionnaire**	Yes	Yes	Yes	Yes	Yes	Yes	yes
**Promis 29**	Yes	Yes	Yes	Yes	Yes	Yes	Yes

*ASES* American Shoulder and Elbow Surgery score, *SANE* Single Assessment Numeric Evaluation score, *ROM* range of motion, *Promis 29* Patient-Reported Outcomes Measurement Information System version 2.0

### Statistical analysis

#### Sample size

To determine sample size of this parallel design, the study was powered (a-priori) for the primary outcome measure of the American Shoulder and Elbow Surgeon (ASES) scale at baseline, 2 and 6-weeks, 3 and 6 months post replacement. Study assumptions estimate a 12 month ASES score of 79/100 for the PT group (SD = 15.0) and 69/100 for the HT group (SD = 15.0). The 10 point change is considered a clinically meaningful difference in groups [10].

Assuming normal distributions among the 2 independent group ASES measures, and assuming small-to-medium effect based on between-group differences, we constructed a sample size estimation using a Repeated Measures Analysis of Covariance (ANCOVA). Measuring fixed effects, main effects, and interactions, with an expected 80% power, 6 dedicated time intervals (including baseline), two independent groups, 2 covariates, 1 outcome measure, and a standard error of probability of 0.05, we estimate the need for a minimum sample size of 158 for statistical significance (~ 79 per group). To account for the expected potential 24 crossover (15%) subjects, we will increase enrollment to 182 total. Missing values will be adjusted using a chains equation, multiple imputation method in which we will assign predictor, structural and impute variables.

#### Data analysis primary outcomes

Study data are collected by clinical research assistants and managed using REDCap electronic data capture tools at the lead institution site (Rush University Medical Center). REDCap (Research Electronic Data Capture) is a secure, web-based software platform designed to support data capture for research studies, providing 1) an intuitive interface for validated data capture; 2) audit trails for tracking data manipulation and export procedures; 3) automated export procedures for seamless data downloads to common statistical packages; and 4) procedures for data integration and interoperability with external sources [36].

Chi square and t-tests will be used to measure differences in baseline characteristics of both groups. Linear mixed effects modeling will be used to measure differences in the outcomes associated with ASES scores, pain, SANE, and range of motion measures across all time points. Linear mixed effects modeling is robust to individual changes, allows for control of variables that are different at baseline, and allows for control of variables that influence outcomes. Linear mixed effects modeling is not bound by the same assumptions as OLS modeling methods (e.g., ANOVA). Differences will be captured for up to 6 time points (pre op to post at 2 and 6 weeks, 3, 6, 12 and 24 months). A *p* value of < 0.05 will be considered statistically significant for all analyses.

We will evaluate the primary outcomes with two sets of statistical analyses: One with intention to treat including patients who crossed over and a second adjusted to the cross-overs of the patients. Non-adherence to randomly assigned treatments can often mean that the intention-to-treat analysis underestimates the real benefit of the treatment [48]. We will run a preplanned sensitivity analysis evaluating the descriptive statistics of those who crossed over to those who did not. We will also run an estimated “as treated” longitudinal analysis based on comparisons of those actually treated with PT and HT. We will analyze a repeated measures, linear mixed effect model in which our outcomes will remain the dependent variable and treatment received will be incorporated as a time varying covariate. Adjustments will be made for the time of cross-over with respect to the original enrollment date to approximate the designated follow-up times. Baseline variables that were individually found to predict missing data or treatment received will be included to adjust for possible confounding.

#### Data analysis secondary outcomes

##### Complications analysis

The complication rate is defined as the incidence of the occurrence of the complication divided by the total number of subjects in the PT or HT group, expressed as a percentage. Statistical difference between the two groups will be compared between the PT and HT groups using a Chi Square test with level of significance *p* < 0.05.

##### Quality of life and cost analysis

To analyze quality of life and cost-effectiveness differences between the PT and HT groups, two formal steps will be followed. Step one will involve a crosswalk of PROMIS 29.2 measures to EQ5D quality of life measures, [49] which is a standardized utility measure. Utilities are measured on a cardinal scale of 0–1, where 0 indicates death and 1 indicates full health. Using the ‘anchors’ of 0 and 1, utility measurement is on an interval scale, where the same change means the same irrespective of the part of the scale being considered (e.g. a change in health from 0.2 to 0.3 is equivalent to a change from 0.8 to 0.9). States worse than death can also be accounted for, with such states taking a negative value [50]. We will use the formula by Revicki and colleagues to complete the crosswalk [51].

*EQ5D = (1.0266+0.0077) X (PROMIS physical functioning t score -0.0021) X (PROMIS fatigue t score – 0.0040) X (PROMIS pain interference t score – 0.0023) X (PROMIS anxiety t score-0.0022) x (PROMIS depression t score)* [49]

The formulaic calculations will result in a quality -adjusted life year value (QALY). QALY’s reflect two key elements—health related quality of life and survival. QALYs can be aggregated across individuals, i.e., a QALY is a QALY regardless of who gains/loses it. Differences in the QALY between the PT and HT groups will be assessed using a Mann Whitney U test with a level of significance considered *p* < 0.05.

Step two will involve cost-effectiveness of the interventions. Cost effectiveness studies usually provide the result in the form of an incremental cost-effectiveness ratio. Total charges for PT and HT groups will be estimated using mean charges for the billable units at each of our collection locations. Total charges of care will be calculated against the QALY identified in step one to determine the cost of 1 QALY using the modeling method of Riesco-Martínez et al. [52] For example, if the average total cost of PT is $10,000 USD, and the average QALY for patients receiving PT was 0.79, the total costs for 1 QALY would be calculated as $10,000 USD / 0.79 (average QALY), or $12,658 USD/QALY. We will compare PT charges to the HT group charges using a Mann Whitney U test with level of significance of *p* < 0.05.

## Discussion

Currently there are two published studies, which prospectively evaluate rehabilitation following RTSA. Hagen et al. have reported results from a randomised controlled blinded study regarding following RTSA assessing early versus delayed mobilization, and conclude that early mobilization is safe, and may offer advantages to the elderly population [31]. Lee et al. reported that not using a sling following RTSA resulted in non-inferior outcomes as compared to sling immobilization for 3 or 6 weeks [32]. Whilst these studies illuminate the potential benefit of an early and accelerated rehabilitation strategy, they do not address the question of how the rehabilitation should be structured, specifically whether formal physical therapy is required or if the rehabilitation can be conducted effectively through a surgeon directed home exercise program. An ideal rehabilitation strategy following RTSA optimizes outcomes while minimizing cost and inconvenience to patients. Thus, the necessity for formal, clinic-based physical therapy should be determined. This study proposes to answer this question with a large prospective, multi-center, randomised controlled trial across several regions of the United States producing generalizable results. If the HT group has the same or better outcomes than the formal PT group, then surgeon-directed home therapy is a convenient and cost-effective rehabilitation strategy for patients who have undergone RTSA. If the PT group has better outcomes without increased harm/complications to patients, then a studied rehabilitation strategy can be endorsed. The prospective and randomised nature of the project are strengths, whilst the cross-over option allows for patient preference and safety.

One limitation of the project is that all participating surgeons are fellowship trained shoulder surgeons and therefore have special training in performing RTSA. Therefore, results of the surgery and instruction in a home therapy program may not be generalizable to all orthopedic surgeons. A second limitation is that there may be variability of outcomes introduced by therapists in out-patient clinics drifting from the provided rehabilitation guidelines. An attempt at fidelity to the physical therapy intervention is attempted by providing rehabilitation guidelines and therapist training instruction booklets to all treating therapists; however, PT interventions for each patient are not being individually monitored to discern how closely therapists follow the rehabilitation guidelines. However, this somewhat pragmatic approach to out-patient physical therapy renders the results for this group generalizable. A limitation of the HT group is that the method of delivery of instruction of the exercises by surgeons and clinical research assistants at post-operative visits may vary across sites which could introduce variance.

In summary, this project will compare clinical and patient reported outcomes, costs and complications for PT versus HT over a two-year period following RTSA to aide in determining the optimal safe and value-based rehabilitation strategy.

## Supplementary Information

**Additional file 1.** Appendix A. Patient questionnaire.

**Additional file 2.** Informed Consent Form used at the lead institution.

**Additional file 3.** SPIRIT Checklist.

## Data Availability

Not applicable.

## Abbreviations

RTSA: Reverse total shoulder arthroplasty
PT group: Physical therapy group
HT group: Home therapy group
ASES: American Shoulder and Elbow Surgeons
SANE: Single alpha-numeric evaluation
OA: Osteoarthritis
ATSA: Anatomic total shoulder arthroplasty
SPIRIT: Standard Protocol Items Recommendations for Interventional Trials
REDCap: Research Electronic Data Capture
PROMIS: Patient-Reported Outcomes Measurement Information System
ER0: External rotation at 0 degrees of abduction (arm at the side)
ER90: External rotation at 90 degrees of abduction
IR: Internal rotation
ICC: Inter-tester correlation coefficient
ICM: International Consensus Meeting
ANCOVA: Analysis of covariance
QALY: Quality-adjusted life years
