# Supplementary material for: Protocol for a multicenter, randomised controlled trial of surgeon-directed home therapy vs. outpatient rehabilitation by physical therapists for reverse total shoulder arthroplasty: the SHORT trial
Source: Arch Physiother. 2021 Dec 10;11:28. doi: 10.1186/s40945-021-00121-2 (PMC8662891; doi:10.1186/s40945-021-00121-2)
Supplement: Supplementary file 1 — Additional file 1. Appendix A. Patient questionnaire. [file 40945_2021_121_MOESM1_ESM.pdf]

Study Questionnaire

Site ID	Subject Initials	Subject ID

Initials of Tester_________ Call Date _______________

Time: [__] 2wks [__] 6wks [__] 3mo [__] 6mo [__] 1yr [__] 2yr

PAIN QUESTIONS

1. In the last 2 weeks, how much pain from 0-10 have you had in your operative shoulder where 0 is no pain at all, and 10 is the worst pain you can imagine?
   Lowest pain (0-10) [_____]
   Highest pain (0-10) [_____]
   Overall pain (0-10) [_____]

2. SPECIFICALLY FOR YOUR OPERATIVE SHOULDER, over the past 2 weeks, how often have you needed the following medications?

	Never	Occasionally	Daily or near daily	How long have you been taking this medication?
Tylenol (Acetaminophen)				
Anti-inflammatory medications (eg. Motrin, Advil, Naprosyn, Aleve, Aspirin, Celebrex)				
Stronger pain medication (eg. Codeine, Percocet, Dilaudid, Oxycontin, Darvon, Morphine, MS-Contin, Vicodin, Ultram)				
Other (state what other meds are using for pain):				

3. How would you rate your shoulder today as a percentage of normal on a 0-100% scale with 100% being totally normal.
   [______]

THERAPY QUESTIONS

4. Were you scheduled to see the physical therapist since your last follow up?
   - ☐ No *(skip to question 7)*
   - ☐ Yes

5. Since your last follow up, how many days were you scheduled to see the physical therapist?
   [_____] days

6. How many days did you actually go to physical therapy?
   [_____] days

7. How many days did you miss physical therapy?
   [_____] days

8. How did you get to your physical therapy appointment?
   - ☐ Drove myself
   - ☐ Family or friend drove me
   - ☐ Public transportation
   - ☐ Paid transportation (Uber, taxi…)
   - ☐ Other (Please specify):____

9. If you missed therapy, what was the reason for missing:
   - ☐ Too much pain
   - ☐ Sick/illness
   - ☐ Lack of transportation
   - ☐ Forgot about the appointment
   - ☐ Other (please specify):_______

10. Since your last follow up, how many days did you do ALL of your prescribed home exercises?
    [_____] days

11. Since your last follow up, how many days did you do PART of your prescribed home exercises?
    [_____] days

12. Since your last follow up, how many days did you do NONE of your prescribed home exercises?
    [_____] days

13. If you missed or only did part of your prescribed home exercises, what is/are the reason(s) *(check all that apply):*
    - ☐ I was in too much pain
    - ☐ I was too busy to do them
    - ☐ I don’t think they are helping me
    - ☐ I forgot
    - ☐ Other (specify):____

COMPLICATIONS QUESTIONS

14. Since your last follow up, have you developed any of the following problems:
    - ☐ Sharp pain on the top of your shoulder?
    - ☐ Excessive muscular soreness in your arm?
    - ☐ Dislocation/instability of the shoulder requiring you to go the doctor?
    - ☐ Fracture requiring you to go to the doctor?
    - ☐ Infection requiring you to go to the doctor?

15. Since your last follow up, did you have to see a doctor about your operative shoulder for any reason other than those mentioned in prior question?
    - ☐ No *(skip to OTHER HEALTH CONSIDERATIONS)*
    - ☐ Yes

    If so, what was the reason?____

16. If the answer to the above question was yes, what doctor did you see?
    - ☐ Same orthopedic surgeon who referred you to the study
    - ☐ Different orthopedic surgeon
    - ☐ Primary Care Physician
    - ☐ Rheumatologist (arthritis doctor)
    - ☐ Emergency Room personnel
    - ☐ Nurse practitioner or Physician Assistant

OTHER HEALTH CONSIDERATIONS

17. Have you developed any of the following problems since the study started?
    - ☐ Heart attack
    - ☐ Heart failure
    - ☐ Cancer
    - ☐ Fracture (other than the operative shoulder)
    - ☐ Diabetes

18. Have you had any of the following since the study started?
    - ☐ Surgery for your other shoulder?
    - ☐ Any other orthopedic surgery?
    - ☐ Any other surgery?
    - ☐ Hospitalization for any other reason?

WORK HISTORY AND RESOURCE UTILIZATION

19. Which of the following best describes your current work situation?
    - ☐ Employed full time
    - ☐ Employed part time
    - ☐ Retired
    - ☐ Not working due to shoulder surgery/recovery
    - ☐ Not working due to other reasons (for example disability, homemaker)

20. Since your last follow up, how many hours did you or another person miss from work because of your shoulder surgery?
    (Include hours missed for sick days, time went in late or left early due to appointments or other reasons related to your shoulder. Do not include time missed to participate in this study).
    [_______]

    Since your last follow up, how many hours did you or another person miss from work because of any other reason (eg. other health concerns, vacation, time off for the study)?
    [_______]

21. Since your last follow up, much has your operative shoulder affected your ability to do your regular daily activities (other than work at a job)? By regular activities we mean the usual activities you do, such as work around the house, shopping, child care, exercising, etc.

    The effect of my operative shoulder on my regular activities has been: (circle number)

0	1	2	3	4	5	6	7	8	9	10
None		Very Small		Small		Moderate		Large		Very Large

22. Since your last follow up, how many days did you cut down on the things you usually do because of your operative shoulder?

    [_______]
