# Supplementary material for: Protocol for a multicenter, randomised controlled trial of surgeon-directed home therapy vs. outpatient rehabilitation by physical therapists for reverse total shoulder arthroplasty: the SHORT trial
Source: Arch Physiother. 2021 Dec 10;11:28. doi: 10.1186/s40945-021-00121-2 (PMC8662891; doi:10.1186/s40945-021-00121-2)
Supplement: Supplementary file 2 — Additional file 2. Informed Consent Form used at the lead institution. [file 40945_2021_121_MOESM2_ESM.pdf]

Site Principal Investigator Name and Title: Grant Garrigues, MD
Department: Orthopedics
Address and Contact Information: 1611 W Harrison St., Suite 300, Chicago, IL 60612, (312) 432-2337

Protocol Title: Multicenter randomized trial of home vs. physical therapy directed rehabilitation for reverse total shoulder arthroplasty

Sponsor: The Orthopaedic Research and Education Foundation (OREF)

Name of Participant: ____

RUSH

CONSENT FOR PARTICIPATION IN A RESEARCH STUDY

Key Information:

You are being invited to participate in a research study. Research studies answer important questions that might help change or improve the way we do things in the future.

This consent form will give you information about the study to help you decide whether you want to participate. Please read this form, and ask any questions you have, before agreeing to be in the study.

Taking part in this research study is voluntary. You do not have to participate in this study and may choose to leave the study at any time. If you decide not to participate in this study or leave the study at a later time, your health care, benefits, or relationship with Rush University Medical Center will not change or be affected.

The purpose of this study is to compare formal physical therapy with home therapy after reverse total shoulder replacement for the following patient outcomes: pain level, range of motion, and patient satisfaction scores.

A second purpose of the study is to determine if participants who have formal physical therapy have a higher occurrence of postoperative complications including fracture (bone breaks) or instability of the shoulder (head of the upper arm bone slips out of place).

If you agree to participate in this study, your participation may last up to 2 years after surgery.

You will be asked to complete standard of care post-operative visits at 2 weeks, 6 weeks, 3 months, 6 months, 12 months and 24 months.

In addition, at the preoperative appointment (screening visit) you will be randomly assigned (by chance, like the flip of a coin) to receive rehabilitation either as a formal clinic based physical

therapy program, or as a home therapy program directed by your surgeon at your routine scheduled appointments beginning at the 2-week visit. Participants who are in the physical therapy group will attend therapy approximately once a week for 3 months at an outpatient clinic beginning approximately 5 days after surgery. This is routine care.

Participants who are in the home therapy group will receive exercise and activity guideline instructions by their surgeon at the routine postoperative clinic visits following surgery. This will begin at the 2-week appointment. This is an alternate routine care.

Both groups will be given a questionnaire at routine post-operative appointments. You will be asked to answer questions about your pain level, complications, adherence (ability to follow directions) to treatment plan, surgical shoulder range of motion, your activity level and satisfaction.

For a detailed description of study procedures, please see the "*What are the activities you will be doing if you participate in this study?*" section of this consent form.

There are risks to you for participating in this study,. This study includes little risk, which means that there is no more expected risk to you than what you might experience during a typical day or during a routine physical exam. There is a risk loss of confidentiality by participating in a research study, but precautions will be taken to prevent this from happening. For details and a list of risks you should know about, please see the "What are the risks and discomforts of participating in this study?" section below.

You may benefit from taking part in this study, but there is no guarantee that it will help you. However, because individuals respond differently to therapy, no one can know in advance if it will be helpful for you.

There are other options available to you if you decide not to participate in this study. Instead of participating in this study, you may choose another form of treatment such as: receiving formal physical therapy or home exercises according to your surgeon's standard practice. You do not have to be in this study to be treated for reverse total shoulder replacement. You should discuss other options with your study doctor.

<u>Detailed Information</u>: Please review the rest of this document for details about the above topics and additional information you should know before making a decision about whether or not you will participate in this study.

<u>Why are you being invited to participate in this study?</u>
You are being asked to participate in this study because you are having a reverse total shoulder replacement surgery.

<u>How many participants will take part in this study?</u>
Approximately 200 participants are expected to take part in this study across all sites. We expect to enroll approximately 80 participants at Rush University Medical Center.

What are the activities you will be doing if you participate in this study?

If you agree to be in this study, you will be asked to participate in the following activities:

- If you agree to be in this study, you will be asked to sign and date this consent form.
- A preoperative appointment as a part of routine care. This will serve as the screening visit.
- At the screening visit, you will be randomly assigned (like the flip of a coin) to receive rehabilitation either as a formal clinic based physical therapy program, or as a home therapy program directed by your surgeon at your routine scheduled appointments. You have a 1 in 2 chance of receiving rehabilitation in the physical therapy group, and a 1 in 2 chance of receiving home therapy.
- Participants will have routine reverse total shoulder replacement surgery as an inpatient at Rush University Medical Center or Rush Oak Park Hospital, and an Occupational Therapist in the hospital will instruct them in their initial exercises prior to discharge as part of routine treatment.
- Participants who are in the physical therapy group will attend therapy approximately once a week for 3 months at an out-patient clinic beginning approximately 5 days after surgery. This is considered routine care.
- Participants who are in the home therapy group will receive exercise and activity guideline instructions by their surgeon and staff at the 2-week routine postoperative clinic visit following surgery. This is an alternate routine care.
- Both groups of participants – physical therapy and home therapy – will be expected to perform exercises at home several times a day, and to follow activity guidelines and precautions associated with reverse shoulder replacement.
- You will be asked to complete standard of care post-operative visits at 2 weeks, 6 weeks, 3 months, 6 months, 12 months and 24 months. As part of your regular standard of care, you will be asked to have certain tests, x-rays, and/or procedures performed. The study doctor will use these test results both to treat you and to complete this research. These test results will be recorded in your medical record and your research record.
- Both groups will be given a questionnaire at routine post-operative appointments. You will be asked to answer questions about your pain level, complications, adherence (ability to follow directions) to treatment plan, surgical shoulder range of motion, your activity level and satisfaction. A study coordinator will visit you during these appointments, which will take approximately 15 minutes to complete in addition to your clinic visit. If you miss a clinic visit, you may be contacted and asked to fill out questionnaires electronically or by mail.

Will you be contacted about participating in future research?

If you agree, we may contact you after your participation in this study about participating in future research. Please initial and date one of the following options:

_______ _____________ Yes, I agree to be contacted about future research.
Initials Date

_______ _____________ No, I do NOT agree to be contacted about future research.
Initials Date

What are the risks and discomforts of participating in this study?

Side effects, risks, and/or discomforts from participation in this study may include:

- The potential risk of loss of confidentiality. Every effort will be made to keep your information confidential; however, this cannot be guaranteed.
- Some of the questions we will ask you as part of this study may make you feel uncomfortable. You may refuse to answer any of the survey questions and you may take a break at any time during the study.
- There are no additional physical risks associated with this study. However, it is not known if there are more or less risks in having physical therapy in the clinic versus home therapy.

There may be other risks that may happen that we cannot predict.

What if there is new information that may affect your decision to participate in this study?
During this study, you will be told about important findings (either good or bad), such as changes in the risks or benefits of participation in the study or new choices to participation that might cause you to change your mind about being in the study. If new information is shared with you, you may be asked to sign a revised consent form in order to continue participating in this study.

Will you receive your individual results from the study?

Generally, activities performed for research purposes are not meant to provide clinical information. We may learn things about you from this study which could be important to your health or treatment. If this happens, this information will be shared with you.

Can you leave or be removed from this study?
You have the right to leave a study at any time without penalty. For your safety, however, you should consider the study doctor's advice about how to leave this study. If you leave this study before the final study visit, the study doctor may ask you to complete the final steps. This includes assessment of your pain level, range of motion, activity level and completion of satisfaction questionnaires.

The researchers and Sponsor also have the right to stop your participation in this study without your consent if:

- They believe it is in your best interests;
- You do not follow the instructions;
- The study is cancelled for any reason.

What about confidentiality of your medical information?

This authorization is voluntary. Rush University Medical Center and its affiliates ("Rush") will not withhold or refuse your treatment, payment, enrollment, or eligibility for benefits if you do not sign this authorization. You do not have to sign this authorization, but that means that you cannot be in the study or receive study-related treatment.

By signing this document, you voluntarily authorize (give permission to) Dr. Garrigues or his study team, and other Rush personnel involved with the conduct and review of this study (which may include off-site personnel) to use or disclose (release) health information that identifies you for the study described in this document.

During the study, Dr. Garrigues or his study team will collect Protected Health Information (PHI) about you for the purposes of this research. PHI is your health information that includes your medical history and new information obtained as a result of this study. Some of this information will come from your medical record. The health information that Rush may use or disclose for this research includes:

- Physical exam data
- Lab test results
- X-rays or other imaging results
- Address
- Dates of treatments or surgeries
- Telephone and/or Facsimile Number
- Email Address
- Social Security Number
- Medical Record Number or Health Plan Beneficiary Number

Dr. Garrigues or his study team may share your health information and the results of your study-related procedures and tests with people outside of Rush who assist with the conduct and review of this study. The persons who receive your health information may not be required by Federal privacy laws to protect it and may share your information with others without your permission, but only if permitted by the laws governing them. Your health information described above may be used or disclosed to:

- To the Researchers
- The study Sponsor: The Orthopaedic Research and Education Foundation (OREF)
- Monitoring agencies such as the Food and Drug Administration (FDA), the National Institutes of Health and the Rush Institutional Review Board (IRB).

While you participate in the study you will have access to your medical record, but Dr. Garrigues is not required to release to you study information that is not part of your medical record. Rush is required by law to protect your health information, and study records that identify you will be kept confidential. The results of study tests/procedures performed as part of this study may become part

of your medical record. Any study information in your medical record will be kept indefinitely. Your identity will not be revealed on any report, publication, or at scientific meetings.

You have a right to inspect and copy the information to be disclosed with this authorization and you may obtain a copy of the information by contacting the office listed below.

If you no longer want to be in the study and do not want your future health information to be used, you may change your mind and revoke (take back) this authorization at any time by writing to Dr. Garrigues at Rush University Medical Center 1611 W Harrison St Chicago, IL 60612. If the authorization is revoked, you will no longer be allowed to participate in the study and previously authorized individuals/entities may still use or disclose health information that they have already obtained about you as necessary to maintain the integrity or reliability of the current study.

This authorization is valid for the entirety of this research study. It will expire upon completion of the study or if you revoke (take back) the authorization.

If you withdraw from this study, the data already collected from you may not be removed from the study records. The study doctor and/or study team may ask you whether they can continue to collect follow-up data on you. If follow-up information will be requested, you will be asked to sign a separate consent form before this information can be collected.

Records of participation in this study will be maintained and kept confidential as required by law.

The Rush Institutional Review Board (IRB) will have access to your files as they pertain to this research study. The IRB is a special committee that reviews new and ongoing human research studies to check that the rules and regulations are followed regarding the protection of the rights and welfare of human participants.

What are the costs to participate in this study?

You or your insurance provider will be responsible and billed for all costs related to your routine medical care for both forms of treatment in this study, including copayments and deductibles. Routine medical care services are those that you would have received for your condition if you were not participating in this research study. Not all services are covered by insurance. Some procedures or scans may require pre-authorization by your insurance plan. We will notify you if we learn that a service is not covered by your insurance plan as part of the pre-authorization process. If it is not covered, you will be responsible for paying for it. The amount of your out-of- pocket expense will depend on your insurance plan.

For beneficiaries with Medicare Advantage Plans, traditional Medicare is billed for the routine cost of a research study. You may have more or higher co-pays than with a Medicare Advantage plan. Please discuss the costs of the study with your surgeon. A Financial Counselor in the clinic can provide you with an estimate of costs for routine services.

We will monitor your RUMC/ROPH patient care charges to make sure that costs are directed appropriately. If you have any questions or concerns about appropriate billing, contact your study

team coordinator so that he/she can help find a resolution.

If you do not have insurance, you will be billed for the amount you have to pay.

Will you be paid for your participation in this study?
You will be paid a $25 gift card at the completion of your 6-month postoperative visit. You will be paid within approximately 30 days of your 6-month postoperative visit in person during your clinic visit or by mail. We may need to collect your social security number or Taxpayer Identification Number (TIN) in order to pay you and for tax reporting purposes to the United States Internal Revenue Service (IRS).

Your participation in this study may contribute to the development of commercial products from which the Sponsor company or others may derive economic benefit. There are no plans to pay you for any of these developments.

What if you are injured as a result of your participation in this study?
If you get ill or injured from being in the study, Rush University Medical Center will help you get medical treatment. You should let the study doctor know right away that you are ill or injured. If you believe you have become ill or injured from this study, you should contact Dr. Garrigues at telephone number 312.432.2880.

You should let any health care provider who treats you know that you are in this study. If you do seek medical treatment, please take a copy of this document with you because it may help the doctors where you seek treatment to treat you. It will also provide the doctors where you seek treatment with information they may need if they want to contact the study doctors.

You or your health insurance plan will be billed. No money has been set aside to pay the costs of this treatment. Health insurance plans may or may not cover costs of research-related injury or illness. You should check with your insurance company before deciding to participate in this research study. Costs not covered by insurance could be substantial.

Rush University Medical Center has no program for financial compensation or other forms of compensation for injuries which you may incur as a result of participation in this study.
By signing this form, you are not giving up any legal rights to seek compensation of injury

Who can you contact for more information about this study?
Questions are encouraged. If you have further questions about this study, you may call Carla Edwards, study coordinator, at 312.947.1881 or email address: carla.edwards@rushortho.com.

Who can you contact if you have concerns about your rights as a study participant?
Questions about the rights of research participants may be addressed to the Rush University Medical Center Office of Research Affairs at 1-800-876-0772.

What are your rights as a study participant?
Taking part in this study is voluntary. If you choose not to participate in this study or to leave the

study at any time, your health care, benefits or relationship at Rush University Medical Center will not change or be affected.

If you choose to leave this study and you do not want any of your information to be used, you must inform Dr. Garrigues in writing at the address on the first page. Dr. Garrigues may still use your information that was collected prior to your written notice.

SIGNATURE BY THE PARTICIPANT

By signing below, you are consenting to participate in this research study. You have read the information given or someone has read it to you. You have had the opportunity to ask questions, which have been answered satisfactorily to you by the study staff. You do not waive any of your legal rights by signing this consent/authorization form. You will be given a signed copy of this document.

______________________	______________________	______________________
Name of Participant	Signature of Participant	Date of Signature

SIGNATURE BY THE INVESTIGATOR/INDIVIDUAL OBTAINING CONSENT:

I attest that all the elements of informed consent described in this consent document have been discussed fully in non-technical terms with the participant. I further attest that all questions asked by the participant were answered to the best of my knowledge.

______________________________________	______________________
Signature of Individual Obtaining Consent	Date of Signature
